# High resolution hemodynamic profiling of murine arteriovenous fistula using magnetic resonance imaging and computational fluid dynamics

**DOI:** 10.1186/s12976-017-0053-x

**Published:** 2017-03-20

**Authors:** Daniel Pike, Yan-Ting Shiu, Maheshika Somarathna, Lingling Guo, Tatyana Isayeva, John Totenhagen, Timmy Lee

**Affiliations:** 1 Department of Bioengineering, University of Utah, Salt Lake City, UT USA; 2 Division of Nephrology and Hypertension, Department of Internal Medicine, University of Utah, Salt Lake City, UT USA; 3 Department of Medicine and Division of Nephrology, University of Alabama at Birmingham, 1720 2nd Ave South, Birmingham, AL 35294-0007 USA; 4 Department of Radiology, University of Alabama at Birmingham, Birmingham, AL USA; 5 Veterans Affairs Medical Center, Birmingham, AL USA

**Keywords:** Computational fluid dynamics, Hemodialysis fistula, Wall shear stress, Magnetic resonance imaging

## Abstract

**Background:**

Arteriovenous fistula (AVF) maturation failure remains a major cause of morbidity and mortality in hemodialysis patients. The two major etiologies of AVF maturation failure are early neointimal hyperplasia development and persistent inadequate outward remodeling. Although hemodynamic changes following AVF creation may impact AVF remodeling and contribute to neointimal hyperplasia development and impaired outward remodeling, detailed AVF hemodynamics are not yet fully known. Since murine AVF models are valuable tools for investigating the pathophysiology of AVF maturation failure, there is a need for a new approach that allows the hemodynamic characterization of murine AVF at high resolutions.

**Methods:**

This methods paper presents a magnetic resonance imaging (MRI)-based computational fluid dynamic (CFD) method that we developed to rigorously quantify the evolving hemodynamic environment in murine AVF. The lumen geometry of the entire murine AVF was reconstructed from high resolution, non-contrast 2D T2-weighted fast spin echo MRI sequence, and the flow rates of the AVF inflow and outflow were extracted from a gradient echo velocity mapping sequence. Using these MRI-obtained lumen geometry and inflow information, CFD modeling was performed and used to calculate blood flow velocity and hemodynamic factors at high resolutions (on the order of 0.5 μm spatially and 0.1 ms temporally) throughout the entire AVF lumen. We investigated both the wall properties (including wall shear stress (WSS), wall shear stress spatial gradient, and oscillatory shear index (OSI)) and the volumetric properties (including vorticity, helicity, and Q-criterion).

**Results:**

Our results demonstrate increases in AVF flow velocity, WSS, spatial WSS gradient, and OSI within 3 weeks post-AVF creation when compared to pre-surgery. We also observed post-operative increases in flow disturbances and vortices, as indicated by increased vorticity, helicity, and Q-criterion.

**Conclusions:**

This novel protocol will enable us to undertake future mechanistic studies to delineate the relationship between hemodynamics and AVF development and characterize biological mechanisms that regulate local hemodynamic factors in transgenic murine AVF models.

**Electronic supplementary material:**

The online version of this article (doi:10.1186/s12976-017-0053-x) contains supplementary material, which is available to authorized users.

## Background

Hemodialysis vascular access dysfunction remains the Achilles heel of the hemodialysis procedure. The arteriovenous fistula (AVF) is the preferred choice of vascular access for hemodialysis patients, but AVF maturation failure remains a critically important clinical problem in end stage renal disease (ESRD) patients on hemodialysis. Up to 60% of newly created AVFs did not successfully mature to become usable [1]. The most common angiographic lesion present in AVF maturation failure is stenosis at the juxta-anastomotic region of the AVF. The two main etiologies of AVF maturation failure are early aggressive neointimal hyperplasia (NH) development and persistent inadequate outward remodeling of the AVF, both of which contribute to formation of venous stenosis at the juxta-anastomotic region of the AVF [2, 3]. AVF maturation failure results in dialysis therapy with a tunneled dialysis catheter or a synthetic AV graft (AVG). Mortality in patients dialyzing with catheters has been reported to be 1.5 times greater than that in patients dialyzing with AVF [4, 5]. When compare to matured AVFs, synthetic AVGs have high failure rates due to stenosis, reported to be 50 and 75% at 1 and 2 years after implantation, respectively [6]. Thus, there is an unmet clinical need to improve our understanding of AVF maturation failure and devise a strategy to improve maturation.

Local wall hemodynamic factors likely play a key role in successful or failed AVF development. The creation of the arteriovenous anastomosis (connection of low-pressure vein to the high-pressure arterial system) results in an immediate increase in blood flow and wall shear stress (WSS) through the AVF inflow artery and outflow vein. Computational fluid dynamic (CFD) modeling of WSS has previously been reported in porcine AVF models [7, 8] and recently in human AVF [9, 10], using non-contrast MRI, computed tomography scans, or three-dimensional ultrasound imaging protocols to obtain information needed for CFD modeling. These previous human and porcine CFD studies had temporal and spatial resolutions of on the order of 5 μm and 1 ms. A murine model has the advantage of readily available genetic manipulation by knockout and overexpression to investigate the mechanisms of AVF maturation failure. This genetic modification cannot be readily performed in porcine or other large animal models. Since murine AVF models are valuable tools for investigating the pathophysiology of AVF maturation failure, there is a need for a new approach that allows the hemodynamic characterization of murine AVF at higher resolutions (on the order of 0.5 μm and 0.1 ms) than those described in the literature. In this manuscript, we report the technical development of non-contrast MRI imaging of a murine AVF model and the subsequent CFD modeling. Our goal is to develop a technique that can be utilized in future transgenic AVF rodent studies in order to elucidate causal mechanisms of AVF failure focusing on local hemodynamic factors that change following AVF creation. To our knowledge, this methods paper is the first to date to present both non-contrast MRI scans and CFD modeling in a murine AVF model.

## Methods

### Surgical arteriovenous fistula creation

All animal studies and experiments were approved by the University of Alabama at Birmingham Institutional Animal Care and Use Committee (IACUC) and performed in accordance with National Institutes of Health guidelines. Our studies utilized male C57BL/6J mice (*n* = 3, Taconic Biosciences, Hudson, NY) aged 8-12 weeks.

**Fig. 1:**
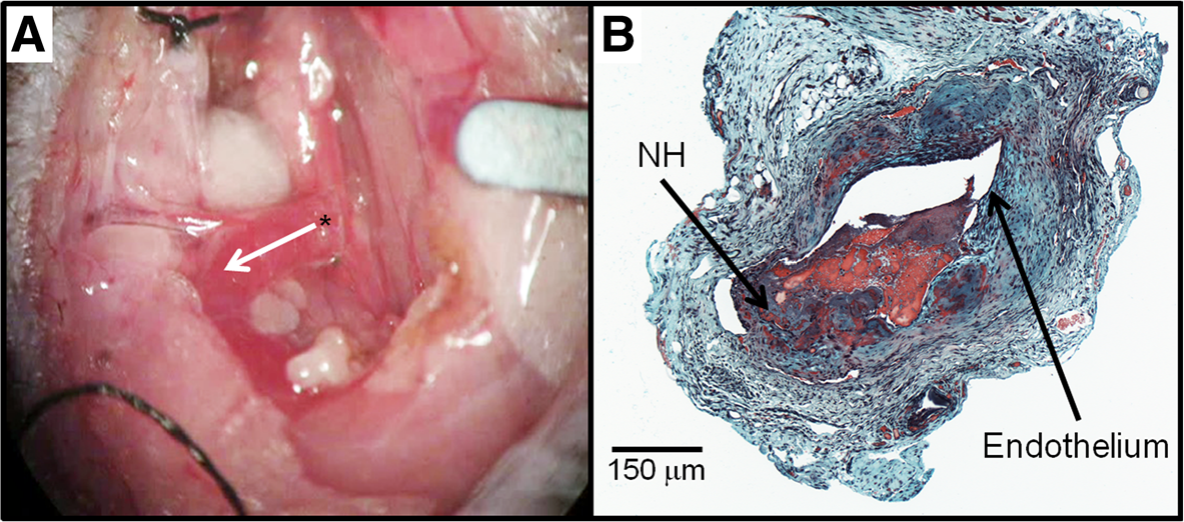
Surgical procedure and histology: (**a**) Arteriovenous fistula (AVF) mouse model using jugular vein (end) to carotid artery (side) configuration. Asterisk (*) depicts the arteriovenous anastomosis. The *white arrow* indicates the direction of blood flow in the venous outflow tract. (**b**) Representative histology of AVF dysfunction (Movat’s stain). Neointimal hyperplasia (NH) was present at 21 Days

After mice (*n* = 2) with AVF were anesthetized with isoflurane, buprenorphine, xyalazine, and ketamine, a midline incision of the surgical area was performed. Using a surgical microscope, the right carotid artery and jugular vein were then exposed. Using 10-0 monofilament microsurgical sutures, a side-to-end anastomosis was created using the carotid artery (side) and jugular vein (end) (Fig. 1a). After unclamping, dilation of the vein and patency was confirmed visually. The mice were maintained on a warming blanket following surgery and buprenorphine was administered two times at 12 hours apart. NH was consistently observed by day 21 post-op (Fig. 1b). The control blood vessels were the pre-surgical carotid artery and jugular vein (*n* = 1), and the contralateral non-surgery carotid artery and jugular vein in the AVF mice at day 7 (*n* = 1) and day 21 (*n* = 1) post-operatively.

### MRI imaging acquisition: time of flight angiography, black blood imaging, and velocity mapping

**Fig. 2:**
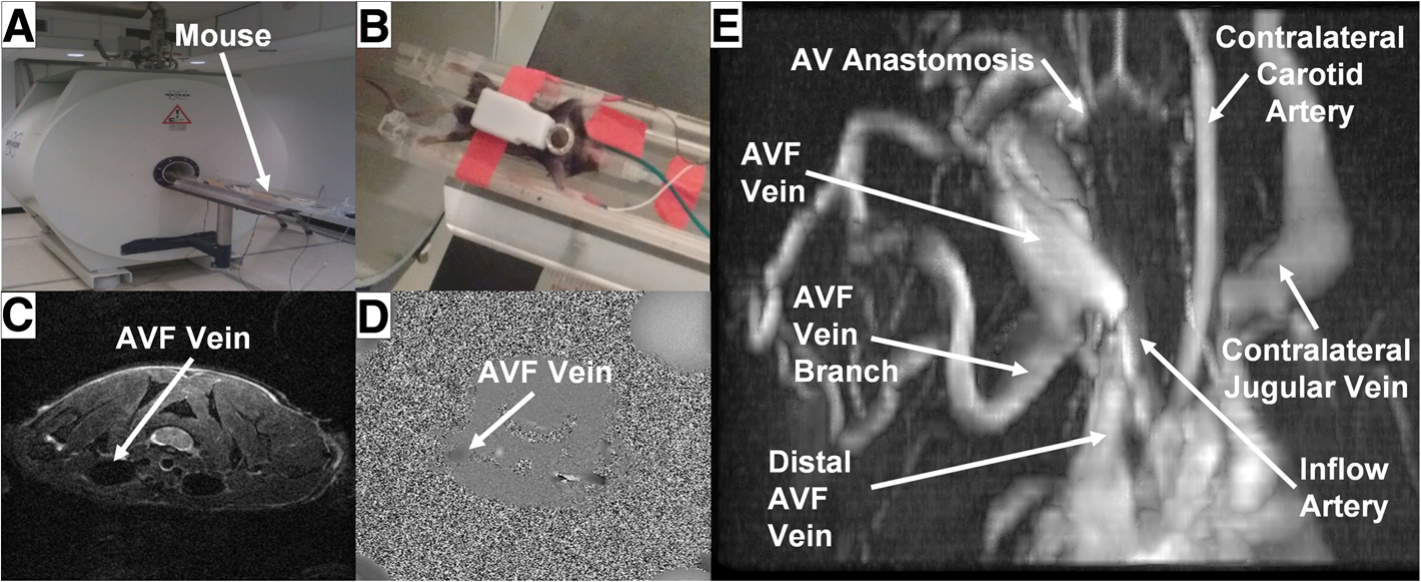
Representative magnetic resonance images of an AVF mouse. (**a**) and (**b**) 9.4 T MRI Imaging of rodents. (**c**) Turbo spin-echo, (**d**) phase contrast, and (**e**) time of flight images

Mice were studied with anatomical imaging and angiography velocity mapping techniques to determine AVF lumen geometry and flow characteristics. Each MRI session lasted approximately 2 hours per animal. During this time, the animal was anesthetized with isoflurane gas at a concentration of 1.5%, and respiration and electrocardiogram (ECG) signals were monitored during MRI acquisition with a physiological monitoring system (SA Instruments Inc., Stony Brook, NY). Mice were imaged in supine or prone position in an animal bed system with integrated tubing for the circulation of heated water (Bruker Biospin, Billerica, MA) to maintain the mouse at 36-38 °C (Fig. 2b).

MRI scanning was conducted using a 9.4 Tesla Bruker Biospec horizontal 20 cm bore instrument with Paravision 5.1 software (Bruker Biospin, Billerica, MA) (Fig. 2a). A 72 mm internal diameter birdcage volume coil was used for signal excitation and a 12 mm diameter surface coil used for reception (Doty Scientific Inc., Columbia, SC).

Scout images were acquired in the coronal, sagittal, and axial dimensions to verify surface coil placement and the location and orientation of the AVF vessel. The scout images were acquired with a T2-weighted RARE (Rapid Acquisition with Relaxation Enhancement) sequence and the following imaging parameters: TR (Repetition Time) 2000 ms, TE (Echo Time) 24 ms, RARE factor 4, 1 average, matrix 128×256, FOV (Field Of View) 25.6 mm×51.2 mm for an in-plane resolution of 0.2 mm. 13, 19, and 25 contiguous 1 mm thick slices were acquired, for coronal, sagittal, and axial orientations respectively.

A 2D time-of-flight angiography sequence based on the FLASH method was used to obtain a global view of the AVF geometry (Fig. 2e) in order to orient the 2D T2-weighted fast spin echo sequence. The following imaging parameters were used: TR 18 ms, TE 4 ms, 8 averages, matrix 171 × 171, FOV 25.6 mm × 25.6 mm for an in-plane resolution of 0.15 mm. 50 overlapping axial slices were acquired with a thickness of 0.5 mm and between-slice spacing of 0.35 mm.

A 2D T2-weighted fast spin echo sequence was used with a black-blood double inversion preparation to reduce signal from the blood within the vessels and allow for better visualization of the vessel lumen. The imaging parameters used were: TR 10000 ms, TE 33 ms, 4 averages, matrix 256 × 256, FOV 25.6 mm × 25.6 mm for an in-plane resolution of 0.1 mm. 35 contiguous 0.5 mm thick axial slices were acquired. This scan was used to create the 3D geometry for CFD simulation, and an example is shown in Fig. 2c.

A gradient echo velocity mapping sequence based on the use of bipolar gradient pulses to produce a flow-dependent signal phase was used to obtain quantitative measures of the blood flow at 3 locations in the vicinity of the fistula (the feeding and draining artery, and fistula vein), and at multiple time points within the cardiac cycle. Respiratory and ECG gating were used to minimize image artifacts due to motion. The imaging parameters used were: TR 15 ms, TE 6 ms, 20 averages, matrix 150 × 256, FOV 15.0 mm × 25.6 mm for an in-plane resolution of 0.1 mm. Velocity maps were acquired at 8 frames during the cardiac cycle, with a 16 ms period between frames. A single 1.5 mm thick slice was collected at each of 3 locations in the vicinity of the fistula. An example was shown in Fig. 2d.

### AVF lumen segmentation, reconstruction, and meshing

3D geometric lumen reconstructions were created from multislice 2D T2-weighted fast spin echo sequences with a black-blood double inversion preparation (as detailed in *MRI Imaging Acquisition* above) using Amira 5.2.1 (Visage Imaging, Inc., San Diego, CA). Image data was segmented manually by intensity thresholding using the blowout tool. The resulting 2D sections were reconstructed to generate a 3D surface in STL format, then smoothed in Amira. The AVF mice (*n* = 2) used for developing this MRI-based CFD approach each had a side branch approximately 4 - 5 mm away from the anastomosis (see “AVF vein branch” in Fig. 2e), and this branch was included in CFD modeling (see *CFD Modeling* below). A high-resolution tetrahedral mesh was created from the STL geometry using Ansys ICEM 15.0, with the number of tetrahedra in the final meshes approximately 1.5 million, with an average length of 0.37 μm. This mesh density was determined as described in the *CFD Modeling* section below.

### Measurement of AVF lumen area

Centerlines of the entire murine AVF lumen, from the final smoothed volumetric mesh mentioned in the previous section, were calculated at 0.1 mm intervals using Vascular Modeling Toolkit (VMTK, www.vmtk.org). Next, lumen areas perpendicular to the centerline of the vessel lumen were calculated using a MATLAB script [9]. The average cross-sectional area for the first 4 mm of AVF vein and inflow artery (starting at the anastomosis and moving toward the heart), as well as the area averaged over a 4 mm segment of the pre-surgical and contralateral non-surgery vessels was calculated along the centerline of the vessel lumen and is used to standardize the regions between vessels for comparison. We chose a 4 mm length because the side branch starts between 4 and 5 mm downstream to the anastomosis, and here we focus on the main and proximal AVF vein.

### AVF blood flow extraction

AVF blood flow velocities were extracted from the gradient echo velocity mapping sequence (as detailed in *MRI Imaging Acquisition* above) using Segment 1.9 R2761 (http://segment.heiberg.se) [11] at three locations in the vicinity of the fistula: the feeding (proximal, inflow) and draining (distal, outflow) artery, and fistula vein.

### CFD Modeling

CFD simulations were performed in ANSYS Fluent 15.0 (ANSYS, Inc., Canonsburg, PA), using the 3D volumetric meshes created previously in the *AVF Lumen Segmentation, Reconstruction, and Meshing* section. The entire simulation domain includes the inflow artery, the outflow artery, the main and proximal AVF vein (the segment of the AVF vein upstream to the side branch), the distal AVF vein (the segment of the AVF vein downstream to the side branch) and the side branch. The inlet boundary condition (inflow artery) and two outlet boundary conditions (the distal AVF vein and the side branch) were prescribed as the pulsatile cross-sectional average blood flow velocity calculated from the *AVF Blood Flow Extraction* section. The remaining outlet boundary condition (outflow artery) was set to zero stress. No-slip boundary conditions were prescribed at the vessel wall. The Reynolds numbers ranged between 3 and 60 in our simulation domain and therefore, the laminar flow assumption was used. The first cell height in our models was prescribed to be 0.1 μm, which was 0.4 – 5.9% of the boundary layer thickness. We also assumed that blood was incompressible and Newtonian, and that the vessel wall was rigid and immobile [9]. With these assumptions, the Navier-Stokes equations reduced to the form in Eq. 1, and the Equation of Continuity reduced to the form in Eq. 2, where ρ is blood density (1050 kg/m^3^), u is blood velocity (m/s), t represents time (s), p is blood pressure (100 mmHg), and μ is blood dynamic viscosity (0.0035 Pa · s).

1 $$ \uprho \left(\frac{\partial u}{\partial t}\kern0.5em +\kern0.5em  u\cdot \nabla u\right)\kern0.5em =\kern0.5em -\nabla p\kern0.5em +\kern0.5em \mu {\nabla}^2 u $$

2 $$ \nabla \cdot u\kern0.5em =\kern0.5em 0 $$

Similarly to a previous study [9], time dependent terms were discretized implicitly with second-order accuracy, while the Navier-Stokes equations were discretized with a second-order upwind scheme. A segregated solver was used to solve the Navier-Stokes and continuity equations, with pressure-velocity coupling defined using the Semi-Implicit Method for Pressure-Linked Equations (SIMPLE) algorithm. All simulations were performed as pulsatile, with approximately 1200 time-steps being used over the length of the cardiac cycle (~120 ms, for a step size of 0.1 ms). Each CFD simulation was run for at least 3 cardiac cycles. All results are from the third cycle. Convergence criteria were set as x-, y-, and z-residuals of 1x10^-5^, and a total residual of 1x10^-5^.

To determine the mesh independence, we performed the simulations of the mouse AVF model (Day 7) at 0.5×10^6^ tetrahedra (average length 0.52 μm), 1.5×10^6^ tetrahedra (average length 0.37 μm), and 15×10^6^ tetrahedra (average length 0.23 μm); the time step was 0.1 ms for all. To determine the time step independence, we performed the simulations of the mouse AVF model (Day 7) at three time steps: 1, 0.1, and 0.01 ms; the mesh density was 1.5×10^6^ tetrahedra for all. Results were considered independent when the differences in the simulation results were <5% between two consecutive simulations. This was achieved between 1.5×10^6^ and 15×10^6^ tetrahedra and between 0.1 and 0.01 ms. Therefore, 1.5×10^6^ tetrahedra and 0.1 ms were chosen for all simulations. As compared to previous CFD simulations for human or pig AVF, our step size is an order of magnitude smaller (0.1 ms vs. 1 ms), and so is our length scale (0.5 μm vs. 5 μm). This difference is critically important for investigating the murine geometry and hemodynamics, which are much different from porcine models or human patients studied previously in the literature [7–10].

### Post-CFD processing for hemodynamic parameters

All hemodynamic parameters were calculated in Tecplot 360 (Tecplot, Inc., Bellevue, WA). The hemodynamic parameters of interest were wall shear stress (WSS) magnitude (Eq. 3), spatial wall shear stress gradient (WSSg) (Eq. 4), oscillatory shear index (OSI) (Eq. 5), vorticity (Ω) (Eq. 6), helicity (Eq. 7), and Q-criterion (Eq. 8), where τ_w,x_, τ_w,y_ and τ_w,z_ represent WSS in the x, y, and z direction, respectively, and V represents the volume in a closed surface. Shear stress was calculated in Tecplot 360 as a function of the velocity at each volumetric mesh node. WSS was then calculated as the root-mean-square average of shear stress at the vessel wall. OSI was calculated from WSS over the cardiac cycle. Vorticity is a derived measurement of the local rotation of fluid flow at a point in a flow field, reported in 1/s and calculated from the spatial velocity gradient at any location in the flow field [12] (Eq. 6). Helicity is a derived measurement of the local ‘twisting’ of flow (Eq. 7). Specifically, helicity measures the local linkage or knottedness of streamlines, either in a right-handed helical pattern (positive) or a left-handed helical pattern (negative) [13]. Q-criterion is a derived measurement used to identify local vortices in a flow field. Q-criterion was calculated from both the vorticity and strain rate (S) within the flow field (Eq. 8). Strain rate is a measurement of the change in deformation (without changing volume) with respect to time. A vortex is defined as a location with positive Q-criterion. This indicates that the magnitude of vorticity (rotation) is greater than shear forces (deformation) at that region. This is characteristic of vortices, which tend to have much greater rotational motion as compared to linear motion (deformation). Negative Q-criterion indicates a non-vortex region; this is not necessarily laminar or undisturbed, but has low vorticity compared to strain rate [14].

3 $$ W S S\kern0.5em =\kern0.5em {\left({\tau}_{w, x}^2\kern0.5em +\kern0.5em {\tau}_{w, y}^2\kern0.5em +\kern0.5em {\tau}_{w, z}^2\right)}^{\frac{1}{2}} $$

4 $$ WSSg\kern0.5em =\kern0.5em {\left({\left(\frac{\partial {\tau}_{w, x}}{\partial x}\right)}^2\kern0.5em +\kern0.5em {\left(\frac{\partial {\tau}_{w, y}}{\partial y}\right)}^2\kern0.5em +\kern0.5em {\left(\frac{\partial {\tau}_{w, z}}{\partial z}\right)}^2\right)}^{\frac{1}{2}} $$

5 $$ O S I\kern0.5em =\kern0.5em 0.5\left(1-\frac{\left|{\int}_{\mathrm{o}}^t\left.{\tau}_w dt\right|\right.}{\int_{\mathrm{o}}^t\left|\left.{\tau}_w\right| dt\right.}\right) $$

6 $$ Vorticity\kern0.5em \left(\varOmega \right)\kern0.5em =\kern0.5em \nabla \kern0.5em \times \kern0.5em  u $$

7 $$ Helicity\kern0.5em =\kern0.5em {\int}_v u\cdot \left(\nabla \times u\right) d V $$

8 $$ Q- criterion\kern0.5em =\kern0.5em \frac{1}{2}\left({\Omega}^2-{\mathrm{S}}^2\right) $$

Each wall surface hemodynamic parameter (WSS, WSSg, OSI) is a variable along the vessel wall both axially and circumferentially; the cardiac-cycle average was calculated for WSS and WSSg, and their values at systole and diastole of the cardiac cycle were also given in the text or figure legends. Values of WSS, WSSg, and OSI were averaged over the first 4 mm of the AVF vein and inflow artery, starting from the anastomosis, as well as averaged over 4-mm segments of contralateral non-surgery vessels. Each lumen volumetric hemodynamic parameter (vorticity, helicity, Q-criterion) is a variable throughout the vessel lumen axially; isosurfaces were calculated at three discrete values (indicated in each figure). The single-color 3D surface (the isosurface) in the vessel lumen represents all points in the vessel lumen with a value equal to the specified number.

## Results

**Fig. 3:**
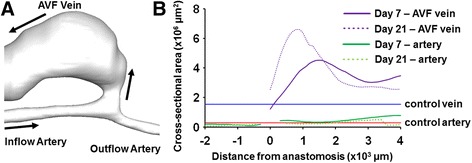
Cross-sectional area of mouse AVF vessels. (**a**) The 3D reconstruction of a representative mouse AVF. Black arrows indicate the direction of blood flow. (**b**) The anastomosis is used as the anatomical landmark and set to be 0. The positive direction is toward the heart, and the negative direction is away from the heart. The *blue line* is the average area of control veins (i.e., pre-surgery and contralateral non-surgery veins): 1.55 ± 0.42×10^6^ μm^2^. The *red line* is the average area of control arteries (i.e., pre-surgery and contralateral non-surgery arteries): 0.29 ± 0.05×10^6^ μm^2^

Figure 3 shows the cross-sectional area of a mouse AVF at day 7 and day 21 post-operatively, as distance from the AVF anastomosis. The cross-sectional area of control vessels (pre-surgery and contralateral non-surgery vessels) is also shown in Fig. 3. We found that, after 21 days, the lumen size of the contralateral non-surgery vessels in the AVF mouse (for the first 4 mm) is similar to pre-surgical size (0.32 ± 0.05×10^6^ μm^2^ pre-surgical artery vs. 0.30 ± 0.04×10^6^ μm^2^ contralateral artery; 2.02 ± 0.29×10^6^ μm^2^ pre-surgical vein vs. 1.77 ± 0.30×10^6^ μm^2^ contralateral vein). On the AVF side (for the first 4 mm), the inflow artery area at both 7 and 21 days post-operatively (0.51 ± 0.15×10^6^ μm^2^ on day 7; 0.31 ± 0.12×10^6^ μm^2^ on day 21) is similar to control arteries, whereas the AVF vein has a trend of increased cross-sectional area within 4 mm proximal to the anastomosis (3.39 ± 0.80×10^6^ μm^2^ on day 7; 3.90 ± 1.44×10^6^ μm^2^ on day 21). Further, this increase was not homogenous along the main and proximal AVF vein (Fig. 3). The increase adjacent to the anastomosis was small, which was likely a result of the restriction of the suture. Lumen expansion at 1 mm was the largest, and then the expansion became smaller. This heterogeneous lumen expansion is also common in human AVFs [9].

**Fig. 4:**
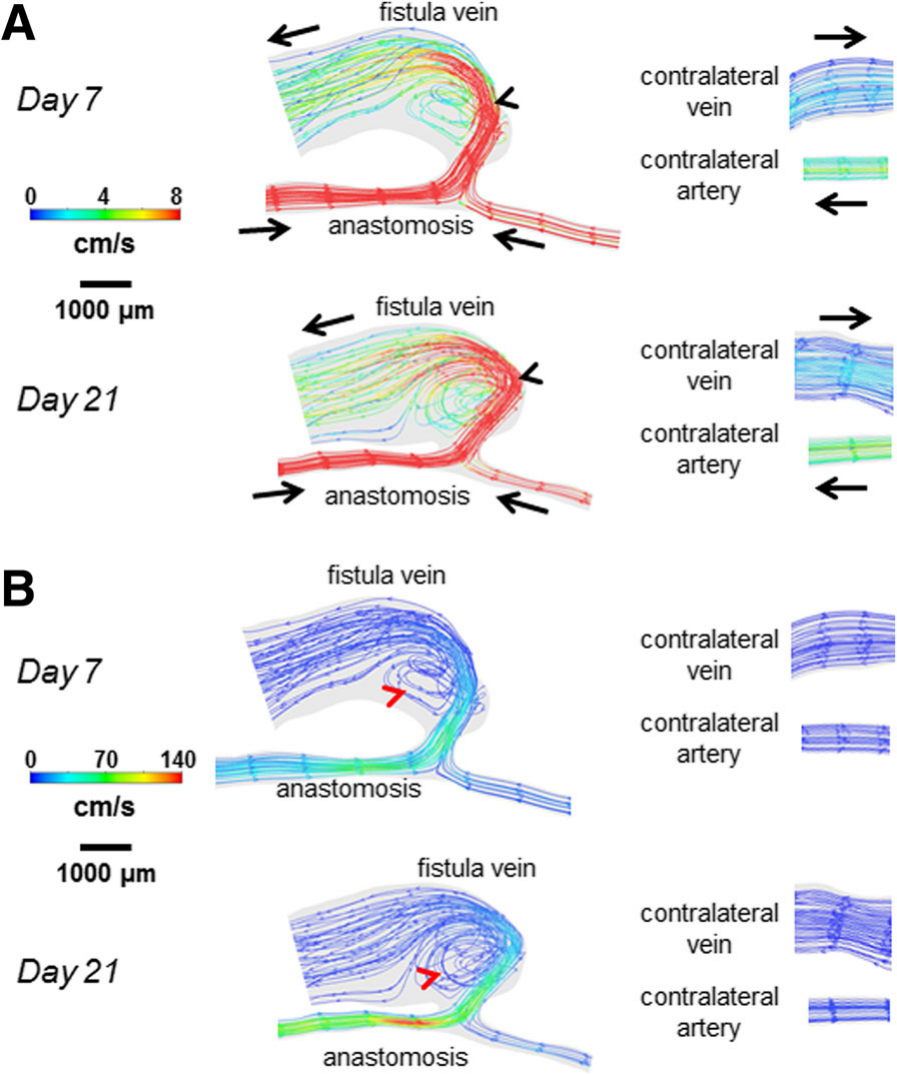
Velocity streamlines for the AVF and contralateral non-surgery controls. The color bars are adjusted to emphasize the velocity distributions in the vein (**a**) and artery (**b**). The velocity was averaged over a cardiac cycle. *Black arrows* indicate the direction of overall blood flow. *Black arrow* heads indicate flow jet into the AVF vein, with velocity of this flow jet increasing from day 7 to day 21. *Red arrow* heads indicate recirculating disturbed flow in the AVF vein near the anastomosis

**Fig. 5:**
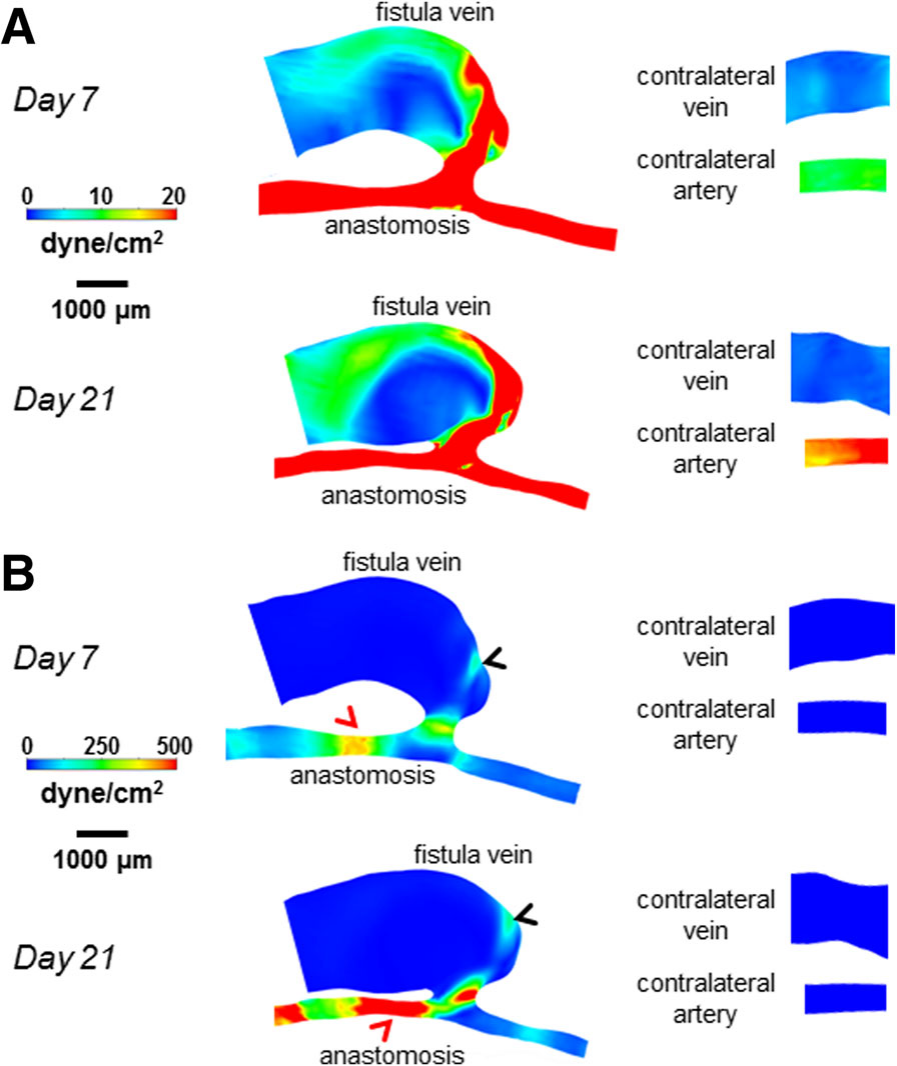
WSS color maps for the AVF and contralateral non-surgery controls. The color bars are adjusted to emphasize the WSS distributions in the vein (**a**) and artery (**b**). The WSS on the maps was averaged over a cardiac cycle. Peak WSS in the AVF vein (*black arrow heads*) was approximately 200 dyne/cm^2^, while peak WSS in the inflow artery (*red arrow heads*) was approximately 500 dyne/cm^2^

**Fig. 6:**
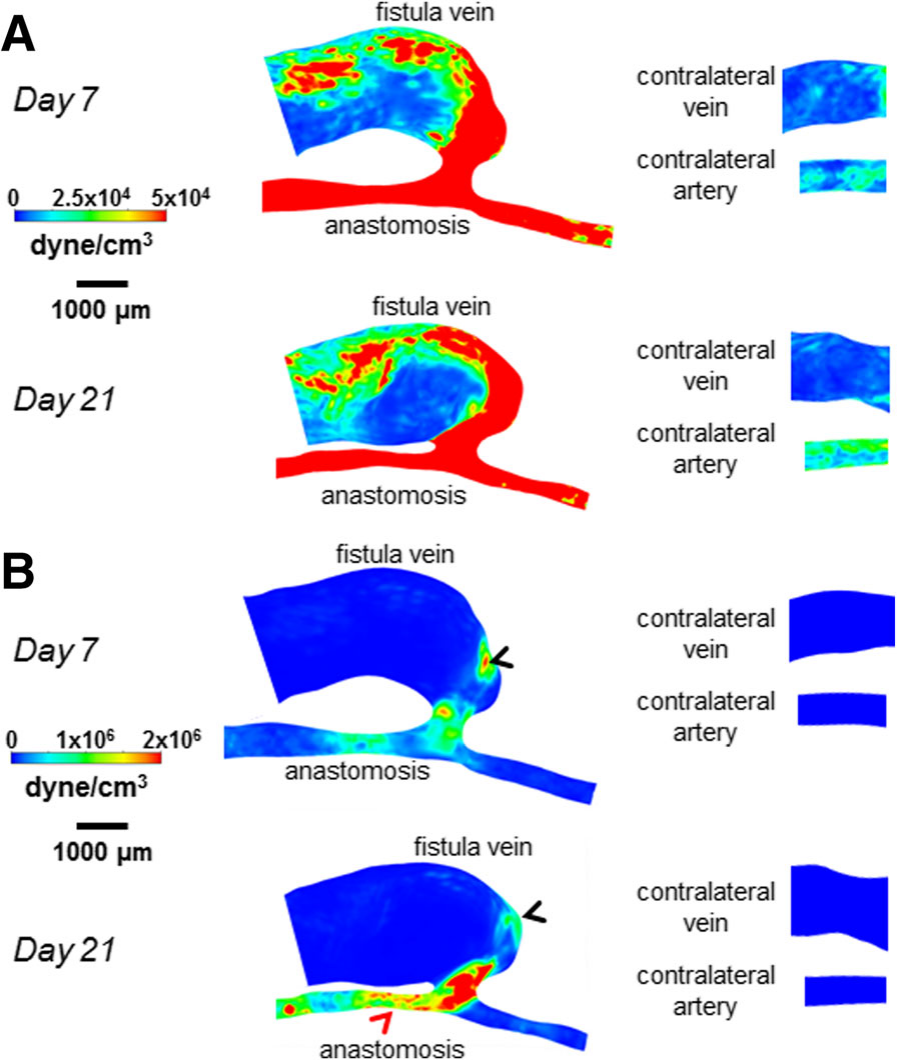
Spatial WSSg color maps for the AVF and contralateral non-surgery controls. The color bars are adjusted to emphasize the WSSg distributions in the vein (**a**) and artery (**b**). The WSSg was averaged over a cardiac cycle. Peak WSSg in the AVF vein (*black arrow heads*) and in the inflow artery (*red arrow head*) was approximately 2×10^6^ dyne/cm^3^

**Fig. 7:**
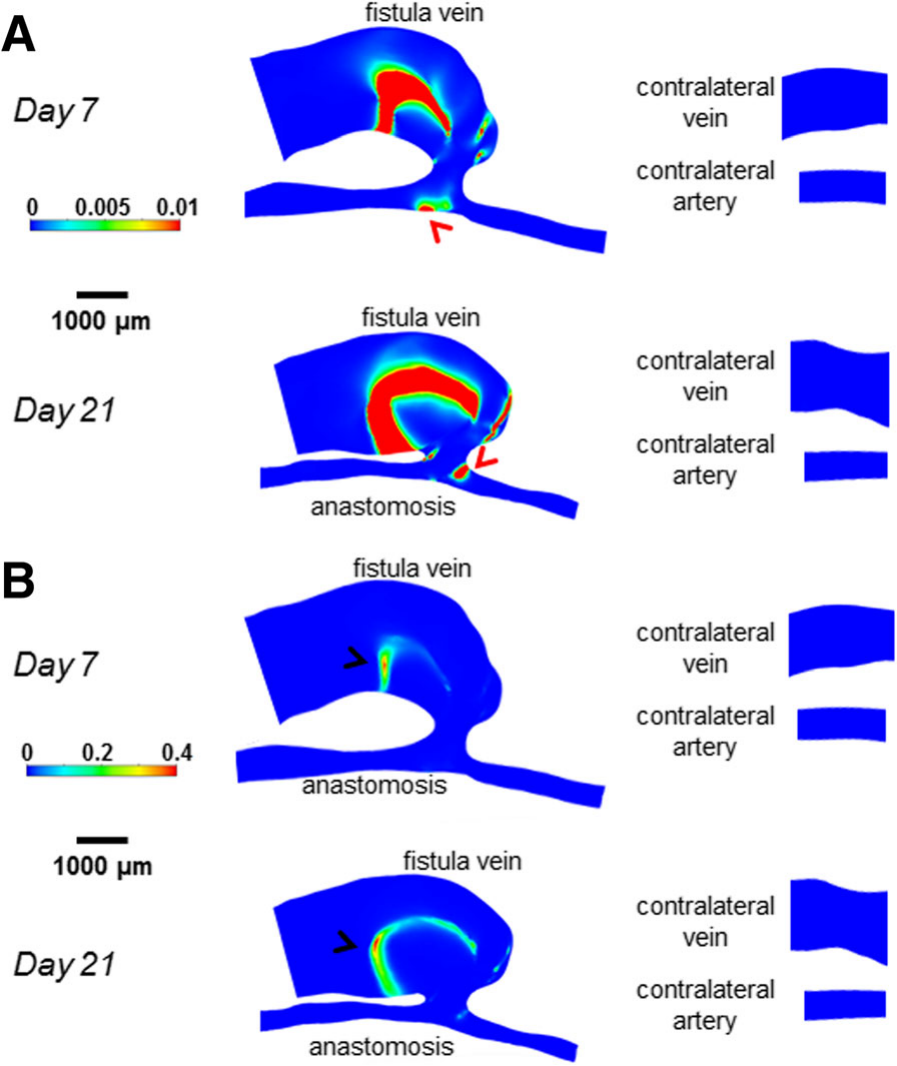
OSI color maps for the AVF and contralateral non-surgery controls. The color bars are adjusted to emphasize the OSI distributions in the vein (**a**) and artery (**b**). Peak OSI in the AVF vein (*black arrow heads*) was approximately 0.4, while peak OSI in the inflow artery (*red arrow head*) was approximately 0.01


Figure 4 shows velocity streamlines, Fig. 5 shows the resulting cardiac-cycle averaged WSS, and Fig. 6 shows the spatial gradient of WSS (WSSg), all of which are averaged over a cardiac cycle. Additional files 1, 2 and 3 show the videos of the time course of velocity streamlines, WSS, and WSSg, respectively, over a cardiac cycle. Figure 7 shows OSI, and Additional file 4 shows the rotated video of OSI.

Additional file 1: Video of day 21 AVF velocity streamlines over a cardiac cycle. The color bars are adjusted to emphasize the velocity distribution in the vein. See Fig. 4a for the labeling of the fistula vein and anastomosis. The velocity plot in the lower left indicates the velocity boundary conditions used in the CFD simulations. (MOV 1698 kb)



Additional file 2: Video of day 21 AVF WSS color map over a cardiac cycle. The color bars are adjusted to emphasize the WSS distribution in the vein. See Fig. 5a for the labeling of the fistula vein and anastomosis. The velocity plot in the lower left indicates the velocity boundary conditions used in the CFD simulations. (MOV 622 kb)



Additional file 3: Video of day 21 AVF WSSg color map over a cardiac cycle. The color bars are adjusted to emphasize the WSSg distribution in the vein. See Fig. 6a for the labeling of the fistula vein and anastomosis. The velocity plot in the lower left indicates the velocity boundary conditions used in the CFD simulations. (MOV 917 kb)



Additional file 4: Video of day 21 AVF OSI color map rotated. The color bars are adjusted to emphasize the OSI distribution in the vein. See Fig. 7a for the labeling of the fistula vein and anastomosis. (MOV 8938 kb)


The AVF vein had increased WSS, WSSg, and OSI as compared to their respective contralateral non-surgery veins (Figs. 5, 6 and 7). Note that this increase is not homogenous throughout the AVF vein. Specifically, at 21 days post-operatively, the velocity, WSS, and WSSg reach peaks of approximately 50 cm/s, 200 dyne/cm^2^, and 2×10^6^ dyne/cm^3^, respectively, in a flow jet along the outer AVF vein wall (Figs. 4, 5 and 6), as compared to peaks of approximately 4 cm/s, 10 dyne/cm^2^, and 2.5×10^4^ dyne/cm^3^ in the contralateral non-surgery veins. Additionally, the expanded AVF vein has distinct recirculating flow (Fig. 4, red arrow head), coinciding with the smaller flow velocity and increased OSI (back arrows in Fig. 7, peaks of 0.4) in that region.

At 21 days post-operatively, when we compare the inflow artery (peaks of approximately 140 cm/s, 500 dyne/cm^2^, 2×10^6^ dyne/cm^3^ and 0.01 for velocity, WSS, WSSg and OSI, respectively) and the contralateral non-surgery artery (peaks of approximately 6 cm/s, 20 dyne/cm^2^, 2.5×10^4^ dyne/cm^3^ and 1x10^-4^ for velocity, WSS, WSSg and OSI, respectively), we found much greater values of all four wall surface hemodynamic factors in the inflow artery. The velocity in the inflow artery is the highest near the AV anastomosis (Fig. 4). When compared to the contralateral non-surgery artery, the averaged WSS and WSSg were higher in the inflow artery (Figs. 5 and 6).

**Fig. 8:**
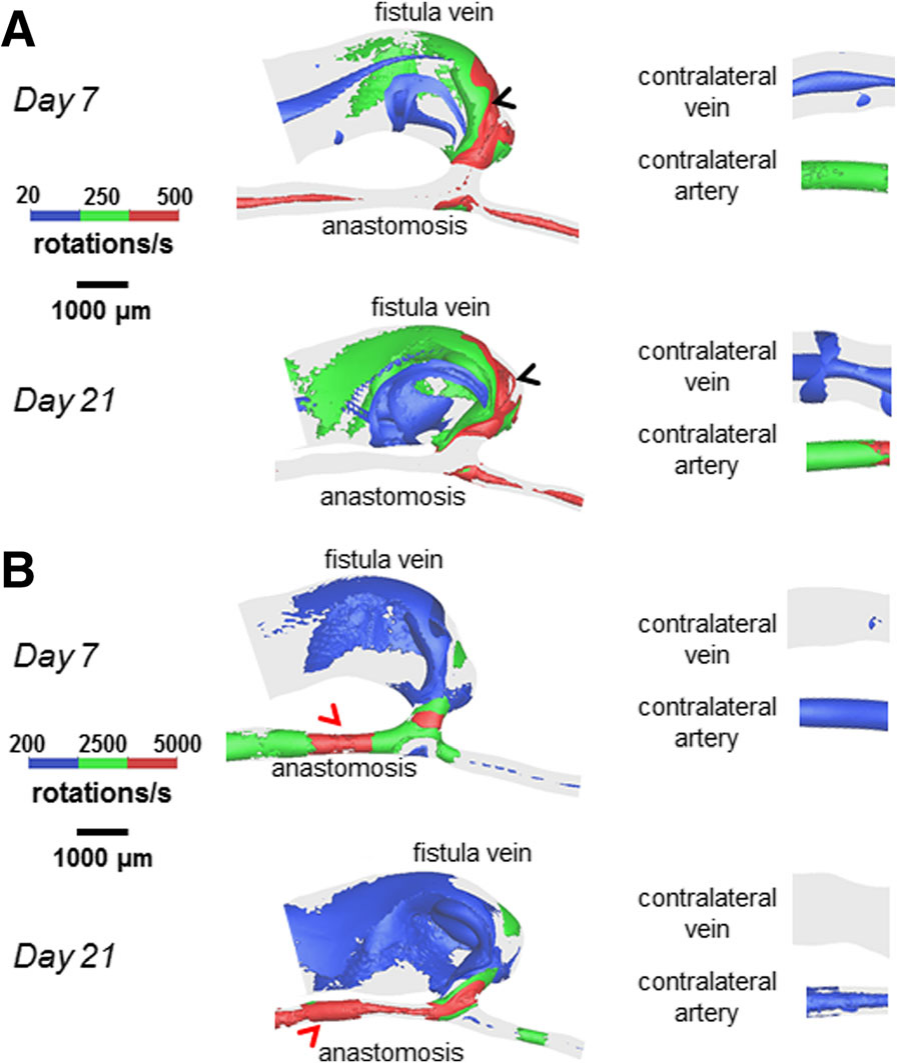
Vorticity isosurfaces for the AVF and contralateral non-surgery controls. The colors are adjusted to emphasize the vorticity distributions in the vein (**a**) and artery (**b**). The AVF vein had substantial regions where vorticity was 500 1/s (*black arrow heads*), and the inflow artery had substantial regions where vorticity was 5000 1/s (*red arrow heads*)

**Fig. 9:**
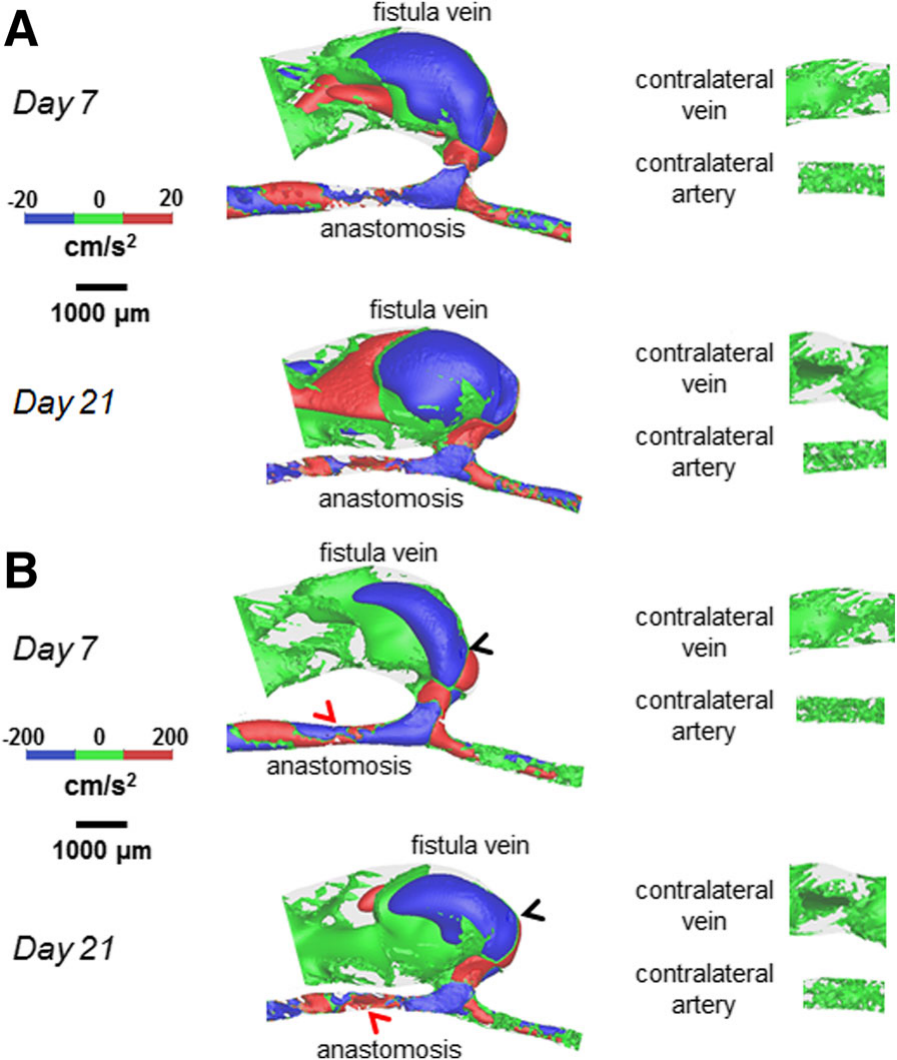
Helicity isosurfaces for the AVF and contralateral non-surgery controls. The colors are adjusted to emphasize the helicity distributions in the vein (**a**) and artery (**b**). Helicity in the AVF vein (*black arrow heads*) and inflow artery (*red arrow head*) had extensive regions equal to ±200 cm/s^2^

**Fig. 10:**
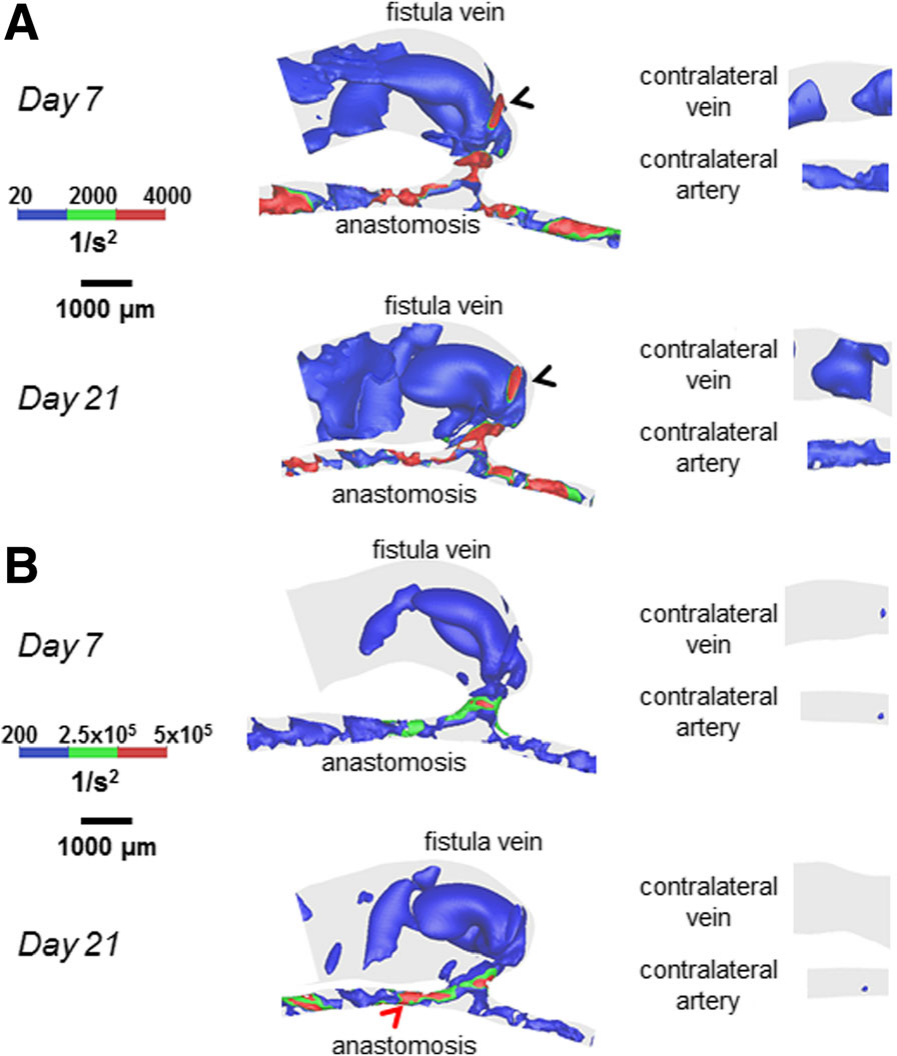
Q-criterion for the AVF and contralateral non-surgery controls. The colors are adjusted to emphasize the Q-criterion distributions in the vein (**a**) and artery (**b**). The AVF vein had regions where Q-criterion was 4000 1/s^2^ (*black arrow heads*), and the inflow artery had regions where Q-criterion was 5×10^5^ 1/s^2^ (*red arrow head*)


Figures 8, 9 and 10 show lumen volumetric hemodynamic parameters (vorticity, helicity, Q-criterion) that are used to describe flow disturbances and vortices. Additional files 5, 6 and 7 show the rotated videos of vorticity, helicity, and Q-criterion, respectively. At 21 days post-operatively, increased vorticity is clear in the AVF vein (peaks of 500 1/s, black arrow heads in Fig. 8) as compared to the contralateral non-surgery vein (20 1/s). In the same vein region, helicity (±200 cm/s^2^ in Fig. 9) and Q-criterion (4000 1/s^2^ in Fig. 10) are also elevated as compared to the contralateral non-surgery vein (helicity 0 cm/s^2^; Q-criterion 200 1/s^2^).

Additional file 5: Video of day 21 AVF time-averaged vorticity isosurfaces rotated. The isosurfaces are selected to emphasize the vorticity distribution in the vein. See Fig. 8a for the labeling of the fistula vein and anastomosis. (MOV 8810 kb)



Additional file 6: Video of day 21 AVF time-averaged helicity isosurfaces rotated. The isosurfaces are selected to emphasize the helicity distribution in the vein. See Fig. 9a for the labeling of the fistula vein and anastomosis. (MOV 8693 kb)



Additional file 7: Video of day 21 AVF time-averaged Q-criterion isosurfaces rotated. The isosurfaces are selected to emphasize the Q-criterion distribution in the vein. See Fig. 10a for the labeling of the fistula vein and anastomosis. (MOV 8783 kb)


At 21 days post-operatively, when we compared the inflow artery (vorticity peaks of 5000 1/s; helicity peaks of ±200 cm/s^2^; Q-criterion peaks of 5x10^5^ 1/s^2^) and the contralateral non-surgery artery (vorticity peaks of 500 1/s; helicity peaks of 0 cm/s^2^; Q-criterion peaks of 200 1/s^2^), we found markedly increased values of vorticity, helicity, and Q-criterion in the inflow artery.

## Discussion

Nearly 70% of end stage renal disease patients utilize hemodialysis as their renal replacement modality of choice [15]. This population of patients requires a functional vascular access to obtain successful long-term dialysis therapy. The recommended vascular access for hemodialysis patients is an AVF [16]. However, up to 60% of AVFs created in the United States fail to mature successfully for dialysis use from a published multicenter randomized controlled trial [1]. The pathophysiology of AVF maturation failure remains poorly understood. In a few small clinical studies, hemodynamic changes following AVF creation have been suggested to play an important role in vascular wall remodeling and AVF development, but have not been characterized in fine spatial or temporal details [17, 18]. Murine AVF models allow the opportunity for detailed mechanistic studies of specific signaling pathways involved in AVF development and maturation. Non-contrast MRI-based CFD modeling allows for analysis of WSS and other hemodynamic measures in AVFs, and currently there are no published techniques regarding CFD modeling of AVF blood flow in small animal models. Here we report a detailed protocol and proof of concept for serial assessment of WSS parameters and lumen area change using an MRI-based CFD approach.

In patients, vein side branches (accessory veins) in the AVF vein are common, and may be ligated during AVF creation surgery [19, 20]. We have found that side branches are also common in our murine AVF model, and our MRI-CFD method can characterize hemodynamics in the side branch as well (data not down). The venous side branch is not the focus of our paper, but in the future, our approach could be used to investigate the effect of side branches on AVF flow and development. Because the segment of the AVF vein between the anastomosis and the branch is the most important part of AVF maturation, here we focus our paper on the AVF vein segment proximal to the anastomosis, before the branch.

In the present study, velocity and resulting hemodynamic parameters (WSS, WSSg, OSI) are substantially elevated (as compared to contralateral non-surgery controls) in both the AVF inflow artery and the main AVF vein (Figs. 5, 6 and 7). We also quantitatively described the disturbed patterns (recirculation, vortices) of the flow paths through the lumen by vorticity, helicity, and Q-criterion (Figs. 8, 9 and 10). Disturbed flow patterns have been linked to the development of atherosclerosis [21] and neointimal hyperplasia in AVF [22–24]. Previous AVF CFD studies in the literature have focused primarily on wall hemodynamics and velocity streamlines; we have expanded this present analysis to include the flow patterns.

Regarding wall hemodynamics and velocity streamlines, previous studies have evaluated the hemodynamics and WSS throughout the arterial or venous tree in mice using various models, including aortocaval AVF, carotid-jugular AVF, carotid artery stenosis by external cast, transverse aortic constriction between the right and left carotid arteries, and partial carotid ligation [25–30]. These studies either calculated WSS using an analytical approximation (such as Poiseuille flow) or used CFD at a much larger spatial interval, such as 1 cm averages. In these studies, the magnitude of WSS in the pre-surgical artery and vein ranges from approximately 10-240 dyne/cm^2^ and 8-18 dyne/cm^2^, respectively; the magnitude of WSS in the post-surgical artery and vein ranges from approximately 100-320 dyne/cm^2^ and 15-180 dyne/cm^2^, respectively [25–30]. Since these studies are in various pathological models, the range of WSS is large. However, our results fit within this range, while providing an order of magnitude smaller spatial resolution (0.5 μm vs. 5 μm in human/pig models) for WSS and other hemodynamic parameters that are necessary for murine AVFs. In addition, our 0.1 ms time step over a 120 ms cardiac cycle in mice distinguishes our study from large animal models with much longer cardiac cycles, such as pigs (cardiac cycle ~800-1000 ms, time step 1 ms) [9]. Currently, there is no consensus on the physiologically relevant level of OSI with respect to causing damage to the endothelium, but similar OSI peaks to our mouse AVF vein at Day 7 and Day 21 (0.4, Fig. 7) were seen in the vein wall of a porcine AVF model (>0.3) [8].

In addition to wall hemodynamics, studying blood flow patterns may be important in helping us better understand the pathophysiology of AVF dysfunction. Previous studies [22] have suggested that the locations of stenosis and NH development in AVFs were associated with low and/or oscillating flow. Defining and characterizing this disturbed flow can be achieved in a more quantitative manner by using volumetric flow measurements, such as vorticity, helicity, and Q-criterion. A study using an in vitro model of human AVG reported directional vorticity of ±550 1/s at the AVG anastomosis [31], and a CFD simulation of intracranial arterial aneurysms reported directional vorticity of -1000 to +1600 1/s [12]. In addition, in a CFD analysis of blood flow in an outflow cannula for cardiopulmonary bypass, helicity magnitude was reported up to 1.5x10^5^ cm/s^2^ [13]. Increasing positive values of Q-criterion (indicating vortices) have been reported in a CFD model of arterial stenosis, with greater regions of positive Q-criterion throughout the artery volume as the stenosis becomes more constrictive [14]. Comparing these previous findings to our work, we find that similar vorticity was seen in our mouse AVF in 4 mm averages (349 ± 768 1/s at Day 21) compared to human AVF and AVG in the vein (550-1600 1/s) [12, 31]. In our mouse AVF vein at Day 21, we calculated increased helicity (96.4 ± 5460 cm/s^2^) and positive Q-criterion (1.21 ± 100×10^3^ 1/s^2^) when compared to non-surgery veins, indicating helical, disturbed flow, and the formation of vortices in the AVF veins, as seen in vascular studies of helical, disturbed flow in cannulated cardiopulmonary bypass and arterial stenosis [13, 14, 32].

## Limitations

The simulation results have been validated against velocity measurements in this study, but not against pressure measurements. Future studies can involve intravenous pressure probes to validate the pressure in addition to the current validated velocity measurements. In this study, our sample size of mice is small, so we did not perform statistical analysis to identify any association between the CFD results and the formation of neointimal hyperplasia, which will need a larger group of mice. However, the main purpose of this study was to describe and report the novel MRI-CFD methodology in a murine AVF model. Our future studies will implement this methodology in larger number of wild type mice to investigate the relationships between the CFD results and the formation of neointimal hyperplasia. Transgenic mice where AVFs are created will be used in concert to delineate the mechanisms.

## Conclusion

To the best of our knowledge, no previous techniques have been published on MRI-based CFD modeling in a murine AVF model, thus, this study is an important technology and tool to advance in the study of the pathobiology of AVF development. Using this non-contrast MRI sequence as the basis for CFD modeling allows for greater spatial and temporal characterization of the resulting hemodynamics. This detail allows for greater insights into the successful or unsuccessful maturation of AVF, particularly in the hemodynamic conditions that lead to pathologic changes such as NH development and resulting stenosis. In the future, our CFD studies will be enhanced in the setting of transgenic AVF mice and complemented with the addition of histological analysis, which can be used to correlate hemodynamic parameters at early time points with NH development and wall thickness changes in the regions of lumen narrowing at later time point.

We have developed a novel approach and method for imaging AVFs created in mice and characterizing AVF flow at high resolutions. Our high spatial and temporal resolution MRI imaging and CFD modeling protocol allows for calculations of WSS, WSS gradients, OSI, as well as quantitation of the complex blood flow patterns by using vorticity, helicity, and Q-criterion. These high quality protocols and tools are currently being used to study hemodynamic wall changes in an expanded research study of transgenic mice with AVFs created to elucidate causal mechanistic changes related to pathways that regulate AVF remodeling in murine AVF models.

## Abbreviations

AVF: Arteriovenous fistula
AVG: Arteriovenous graft
CFD: Computational fluid dynamics
ECG: Electrocardiogram
ESRD: End stage renal disease
FOV: Field of view
IACUC: Institutional Animal Care and Use Committee
MRI: Magnetic resonance imaging
NH: Neointimal hyperplasia
NIDDK: National Institutes of Diabetes, Digestive and Kidney Diseases
OSI: Oscillatory shear index
RARE: Rapid acquisition with relaxation enhancement
SIMPLE: Semi-Implicit Method for Pressure-Linked Equations
VMTK: Vascular Modeling Toolkit
WSS: Wall shear stress
WSSg: Spatial wall shear stress gradient
