# Correction to: High resolution hemodynamic profiling of murine arteriovenous fistula using magnetic resonance imaging and computational fluid dynamics

**DOI:** 10.1186/s12976-019-0104-6

**Published:** 2019-04-09

**Authors:** Daniel Pike, Yan-Ting Shiu, Maheshika Somarathna, Lingling Guo, Tatyana Isayeva, John Totenhagen, Timmy Lee

**Affiliations:** 1 Department of Bioengineering, University of Utah, Salt Lake City, UT USA; 2 Division of Nephrology and Hypertension, Department of Internal Medicine, University of Utah, Salt Lake City, UT USA; 3 Department of Medicine and Division of Nephrology, University of Alabama at Birmingham, 1720 2nd Ave South, Birmingham, AL 35294-0007 USA; 4 Department of Radiology, University of Alabama at Birmingham, Birmingham, AL USA; 5 Veterans Affairs Medical Center, Birmingham, AL USA

**Correction to: Theor Biol Med Model (2017) 14: 5.**

**https://doi.org/10.1186/s12976-017-0053-x**

After publication of the original article [1], the authors have notified us that the name of one of the software in this paper was inadvertently wrong. Specifically, the name cited for the software used in the published version for flow extraction was “Segment 1.9”, instead it should be “ImageJ”.

- The Methods section has therefore been corrected to:

AVF blood flow velocities were extracted from the gradient echo velocity mapping sequence (as detailed in MRI Imaging Acquisition above) using **ImageJ (U.S. NIH, Bethesda, MD) by following similar procedures described previously** [11] at three locations in the vicinity of the fistula: the feeding (proximal, inflow) and draining (distal, outflow) artery, and fistula vein.
